# “Work does change lives”: a qualitative study exploring employment specialists’ perspectives on assisting youth through supported employment in rural Canada

**DOI:** 10.3389/ijph.2026.1609182

**Published:** 2026-08-27

**Authors:** Cassia Warren, Kirsten McCulloch, Jessica Su, Matthew Wenger, Skye Barbic

**Affiliations:** 1 Providence Health Care, Vancouver, BC, Canada; 2 Department of Occupational Science and Occupational Therapy, University of British Columbia, Vancouver, BC, Canada

**Keywords:** employment, individual placement and support, northern Canada, rural, youth

## Abstract

**Objectives:**

To understand the strengths and limitations of implementing supported employment for youth (ages 16–24) in rural northern Canadian communities from the perspective of employment specialists.

**Methods:**

A purposive sample of 10 employment specialists was recruited from Northern British Columbia and interviewed. The qualitative data were analyzed thematically, guided by reflexive thematic analysis.

**Results:**

The analysis showed three themes which can be connected to three levels: (1) Societal factors influencing employment outcomes (macro level), (2) Organizational barriers and facilitators (meso level), and (3) Employment specialists’ capabilities (micro level).

**Conclusion:**

Rural northern communities offer unique strengths (e.g., close-knit social networks, community-driven values) and limitations (e.g., chronic under-resourcing, geographic isolation, access to integrated mental health services). To enhance the success and sustainability of supported employment for youth in rural northern settings, implementation strategies must be tailored to address the intersecting macro (e.g., policy and funding), meso (e.g., organizational capacity), and micro (e.g., individual level) factors that shape program delivery. Strong national and provincial emphasis should be on informing a rural-informed, context-sensitive approach that prioritizes collaboration, long-term investment, and system integration.

## Introduction

Youth in rural and remote regions around the globe face persistent and multifaceted barriers to meaningful and sustained employment [1, 2]. These challenges are often shaped by a lack of access to integrated mental health and social services, geographic isolation, economic precarity, and limited pathways for skill development [1]. The result is that unemployment rates for youth are often two to three times higher than those of adults [3, 4]. Furthermore, rural youth have higher unemployment and lower education rates compared to urban youth [5]. Supported employment (SE) models, particularly those grounded in person-centered, recovery-oriented principles, have emerged as promising interventions to address these barriers, especially for individuals with mental health and psychosocial challenges [6]. Among them, Individual Placement and Support (IPS) stands out as a well-established model designed to provide timely, integrated employment and mental health services [7].

Research has been conducted to understand the implementation of SE programming, such as IPS, in rural settings. Al-Adbulmunem [8] found that challenges to implementing IPS in rural communities in the United States included a lack of employment and workforce options. Moe [9] found that, when examining the implementation of IPS in rural Norwegian communities, employment specialists were heavily car-dependent, driving long distances in between seeing clients and returning to the office, with some employment specialists using their vehicles as office spaces, limiting their flexibility. A systematic review of peer-delivered employment interventions in rural Australian communities found that employment outcomes can not only offer economic, cultural, and social boosts to the community but can also improve the mental health and wellbeing of those who successfully obtain employment [10].

While some work has been done internationally to understand the implementation of SE programs in rural areas, there is still a need to understand cross-regional and local needs at a global level. As SE programs in Canada expand into rural areas, our study aims to understand the strengths and limitations of implementing SE programs for youth in rural Canadian regions that reflect many of the social, geographic, and infrastructural dynamics shared by rural regions worldwide [2].

In Canada, income support for unemployed youth is provided through federal and provincial programs. In British Columbia (BC), youth experiencing financial hardship may be eligible for Income Assistance or Disability Assistance through the Ministry of Social Development and Poverty Reduction (MSDPR). However, Income Assistance benefits have been criticized as insufficient to meet the cost of living and remain well below poverty thresholds and living wage estimates in BC [11–13]. These challenges may be amplified in rural and remote communities, where youth often face limited employment opportunities, transportation barriers, and reduced access to education, health, and social services. Consequently, income support alone may be insufficient to promote labor market participation, particularly for youth experiencing mental health challenges. This underscores the potential value of supported employment programs, such as IPS, which integrate employment services with broader health and community support.

## Methods

### Setting

The study took place in British Columbia (BC) and focused on rural Canadian communities, specifically on communities within the Northern Health Authority, where community sizes may range from a few hundred to approximately 80,000 people (Northern Health, 2024). The Northern Health Authority is one of five regional public healthcare authorities in BC and covers an area of approximately 600,000 square kilometers [14, 15]. Northern Health Authority services are offered throughout 32 communities and 55 First Nation communities, serving approximately 300,000 residents [14]. Participants were located in four communities in Northern BC, which are colonially known as Prince George (population 76,708), Terrace (population 12,017), Fort St. John (population 21,465), and Prince Rupert (population 12,300) [3].

### Participants and recruitment

Our study sample consisted of employment specialists with direct experience supporting youth, aged 16–24, including youth with mental health challenges, in obtaining employment in northern BC. Individuals without direct experience working with youth in Northern BC were excluded.

Throughout this article, we refer to participants as employment specialists.

Participants were recruited via email invitations and direct contact with SE services across Northern BC, with some organizations assisting through program managers. Given the small, specialized workforce, recruitment continued until sufficient informational power was reached, defined by the depth, relevance, and richness of the data in relation to the study aims, rather than by numeric saturation alone.

### Data collection

After informed consent was obtained, [blind for review] conducted semi-structured interviews via Zoom between January 2023 and March 2023 using a co-designed interview guide (see Supplementary Material). Interviews were audio recorded and transcribed verbatim. Field notes were taken during the interview process to capture contextual reflections and emerging impressions. Data were stored on secure servers at the [blind for review], and all local data security regulations were followed.

### Researcher positionality

The study was co-designed and analyzed by two occupational science students ([blind for review]), the senior author ([blind for review]) with over 20 years of experience in the SE space as a researcher and Occupational Therapist, and research coordinators [blind for review] who reside in Northern BC and rural Alberta communities, respectively. This study was also designed in consultation with a supported employment specialist in Northern BC and a program lead for an SE service in urban and rural locations in British Columbia, author [blind for review]. These multiple perspectives informed both data interpretation and reflexive engagement with the analysis.

### Data coding and analysis

The data were analyzed using reflexive thematic analysis (RTA) as outlined by Braun and Clarke [16]. The analysis began with an inductive, data-driven coding process in which transcripts were coded line-by-line to generate initial codes grounded in participants’ accounts, without applying a pre-existing theoretical framework. These codes were then iteratively reviewed, grouped, and refined into candidate themes through repeated engagement with the data and team-based discussions with the research team. These discussions involved comparing interpretations, questioning assumptions, and exploring alternative readings of the data, rather than seeking coding consensus or reliability. The codes were then progressively clustered, reviewed, and refined into candidate themes through an iterative and interpretive process. Themes were developed through sustained movement between coded extracts, full transcripts, and emerging analytic ideas, with ongoing refinement to ensure coherence and depth in relation to the research question. In line with reflexive thematic analysis, themes are understood as actively constructed through researcher interpretation rather than passively identified within the data.

In a second analytic stage, the Micro–Meso–Macro framework was introduced as a conceptual lens through which to further interpret and organize the developing themes. Importantly, this framework was not used to generate initial codes or constrain the analysis; rather, it was used to support reflexive interpretation of how barriers and facilitators operated across interacting levels of influence. The Micro–Meso–Macro framework is widely used in occupational science and social work to examine multilevel determinants of participation and service access [17]. NVivo Release 1.0 Pro [18] was used to manage the data.

Themes were actively constructed through iterative engagement with the data, researcher reflexivity, and ongoing discussion among the analytic team, consistent with a constructionist orientation to RTA.

### Rigor and trustworthiness

We pursued various verification strategies to ensure the rigor and trustworthiness of the data. Trustworthiness was supported through multiple strategies consistent with qualitative research principles. Collaborative coding and regular analytic discussions were used to deepen interpretation and challenge assumptions, rather than to establish inter-coder reliability in a positivist sense. Reflexive memoing was maintained throughout the analytic process to document interpretive decisions, emerging insights, and researcher reflexivity. An audit trail was also maintained to enhance transparency in the development of codes and themes. These strategies supported the credibility, dependability, and confirmability of the findings through an explicitly reflexive and interpretive approach.

## Results

We interviewed 10 participants. The majority of the respondents identified as women (n = 9; 90%) and white (n = 9; 90%). The participants’ ages ranged from 28 to 58 years old (n = 8; mean = 38.63; median = 35.5), and their years of experience ranged from 5 to 25 years (n = 8; mean = 11.44; median = 9.0). Two participants did not report their age or years of experience. Their role titles ranged from employment counselors and consultants to specialists to program coordinators and job developers. They will henceforth be described as “employment specialists.” The participants’ work experiences varied from school settings to community-based or WorkBC employment programs (a government-funded employment service in BC). Two participants reported using the IPS model as a guiding model of care; others named principles such as strength-based approaches and competitive employment opportunities that guided their work. Others reported that they did not utilize any guiding frameworks when working with clients.

Through our reflexive thematic analysis, we constructed three themes: Theme 1. *Macro Level: Societal factors influencing employment outcomes*, Theme 2. *Meso Level: Organizational barriers and facilitators*, and Theme 3. *Micro Level: Employment specialists’ capabilities*. These themes represent iterative constructions developed through iterative engagement with the data, capturing how employment specialists made sense of the multi-level influences shaping supported employment practices.

Theme 1 Macro Level: Societal factors influencing employment outcomes.

Theme 1 relates to the societal factors that influence employment outcomes for youth living in Northern BC. Societal factors are factors that cannot be changed by individual employment specialists but have a daily influence on the youth clients and the employment opportunities available to them.

Participants described that in Northern BC there were many opportunities to work in the resource industry, which offers career development and high wages that are not available in southern parts of BC (e.g., Vancouver). However, they noted that these jobs are difficult to maintain and sustain for those facing barriers such as mental health challenges, workplace discrimination, unstable housing, and low socioeconomic status. Participants also expressed that youth’s values differ from those of older generations and that youth are less inclined to work in the resource industry.

“I also do not think that the older generation is passing those values on to the younger generation, because none of my youth clients want to go and work in oil and gas. I mean lots of them will because that's where the money is, and because it's easy for them to get employment… but that's not what they want… and so I find it hard to engage youth in the employment process.” –P6 [SIC]

Other impacts on SE programs in the north include systemic challenges such as housing insecurity, limited transportation options, internet accessibility, and the availability of healthcare resources.

“We do have city transit system but - oh that's another barrier actually - kids under 12 can ride for free on city transit, I would love to see if kids under 18 could ride for free because that would help significantly. A lot of our kids who are trying to get to work in all other places. They try to work around where their home is but quite often there just are not enough opportunities available, so it would be great to see either transit being made available for them or bus passes.” –P10 [SIC]

Participants reported that they had difficulty engaging with clients who had low motivation and emotional regulation skills, which are often associated with untreated mental health challenges. They advocated for client mental health and substance use challenges to be addressed and prioritized in tandem with employment support, but getting young people access to these services is a challenge of its own due to resource shortages.

“The largest barrier for me is that our community lacks so many resources for youth. the biggest thing I'm seeing is the lack of resources for mental health and specifically youth. And you have a 22-year-old, who needs to work because they are no longer living at home, but they have such bad depression or anxiety, and cannot get a counselor because it costs money. It's just a vicious cycle. So then, we're trying to get somebody a job that does not have the functioning capacity to be able to even work.” -P6 [SIC]

Participants agreed that their services are siloed from other healthcare services, and the gap between employment services and specialized mental health services (e.g., counseling) was mentioned frequently. One participant noted that clients must self-refer to counseling instead of being referred by the employment specialist. Participants repeatedly reported long wait times for mental health services, a shortage of mental health workers, youth admittance to the emergency room for psychiatric help, and a lack of variety in therapy modalities in Northern BC that complement employment services.

“So intergenerational trauma is a huge issue here, and we have a lack of mental health supports. Just cannot find people to fill positions. And like wait lists are huge. So even trying to get mental health or addictions support can be really hard for people.” –P9 [SIC]

These participants’ accounts reflect macro-level structural determinants of employment, particularly with regard to the capacity of the broader mental health system within rural and remote communities. The reference to intergenerational trauma, combined with long waitlists and workforce shortages in mental health and addiction services, illustrates how under-resourced systems and historical social inequities shape access to care, including access to both health and social services. These systemic gaps limit individuals’ ability to obtain the timely support needed for employment readiness and stability, thereby influencing employment outcomes at the population level rather than an individual level.

Theme 2 Meso Level: Organizational barriers and facilitators.

The second theme, “Organizational barriers and facilitators,” captures how organizational, service, employer, and support system factors influence SE effectiveness. Participants emphasized that youth often require integrated mental health and social support; however, interdisciplinary collaboration was limited by resource constraints in rural northern communities. When collaboration was possible, participants felt that services were more effective. Family support could facilitate employment when it aligns with the goals of the youth but could become a barrier when it limits the youth’s autonomy.

“Parents can be enablers of certain behavior, or it could be the opposite. Their parents could be pushing them in the door, and they do not want to be there.” –P6

Another subtheme was bridging the gap between employers’ needs and those of youth. Despite having a shortage of employees, participants reported that employers are often not flexible with the needs of youth, such as accommodations, flexible work hours, and non-customer-facing roles. Participants identified that as an employment specialist, they bridge this gap and advocate for youth.

“ If there were opportunities to create positions for people who could not be forward facing. Because that was probably our biggest challenge, when we had youth who could not like be forward facing for one reason or another. And that really just cuts 75% with the opportunities off the table.” –P2 [SIC]

Participants recognized the importance of on-the-job training and searched for these opportunities for youth. One participant advocated for programs that allow youth to attend school and work at the same time. They agreed that these types of programs could help fill labor shortages in their communities, where many jobs require advanced education:

“In [rural community], a lot of employment opportunities require advanced education such as healthcare, education, mental health, right? And I know that Northern Health has started a program where they will hire people on, and then train them at the same time, so they're doing part schooling, part employment, and still getting to make some money while they're going to school to move up, and that's been something that's been, from what I've seen, really successful. […] So I think programs like that, if we could have more of those it would be better. Especially in a community like this, where we do not have people qualified for these positions that are available.” –P9 [SIC]

Participants noted how they had to increase employers’ awareness of the impact these young people could have and the accommodations that would help them thrive. Specifically, they advocated for employers to receive trauma-informed training, gender inclusivity training, and education about youth’s mental health needs. Participants identified a community-level knowledge gap about accessing SE and hiring youth with mental health needs. Stigma exists for youth with mental health challenges, and this was an emphasized challenge within small communities where “everyone knows everyone.” While some employers saw these youth as a risk, the participants saw them as resilient and able to thrive when provided the right environment.

“I wish they'd realize or understand that the people that I work with have made it this far in life. And what are some of the hardest lives I've ever seen, and if they can make it through that, they can make it through any training that they provide to them.” –P1

Participants identified wage subsidies as a key strategy to reduce employer hesitation by lowering the perceived risk associated with hiring youth with limited work experience or additional support needs. They also highlighted the importance of transition support, including paid certifications and post-secondary education subsidies, which should be embedded within community-informed strategies that are responsive to rural northern contexts.

Participants further described how externally imposed funding structures and program requirements shaped service delivery, including eligibility criteria, program timelines, and funding constraints. Facilitators included evidence-based models such as IPS and interdisciplinary collaboration. While employment specialists felt they could influence organizational factors, they emphasized that sustainable change requires collective commitment from employers, funders, families, and health systems.

Theme 3 Micro Level: Employment specialists’ capabilities.

The third theme focused on items within the employment specialists’ capabilities, defined as those actions that the participants took on a daily basis to help youth find employment. Within this theme, the responsibilities of the employment specialists were focused on one-on-one interactions with youth and/or employers.

Participants identified the importance of client-centered practice and reported that they enable youth to make their own choices about work and provide unjudgmental support and unconditional positive regard throughout the process. For example, if youth did not show up to appointments, the participants kept them on as clients and waited until the youth were ready. They acknowledged that youth autonomy can sometimes be a barrier to employment, but it can also be a turning point in a young person’s life.

“It is a cliché, but from our perspective it comes down to not being judgmental, providing empathy, doing your best to provide unconditional positive regard, right? Again, it's a cliche, but meeting them where they're at. So, we will have clients that we will have the same conversation with in different ways over an extended period of time. Really trying to build a relationship. So maybe you miss this appointment. Maybe you've missed the next 3. I'm not gonna close the door on you. I'm not gonna write you off. I understand you've got a lot of things going on. Sometimes we are the only contact youth might have with service providers.” –P4 [SIC]

Participants noted that being an employment specialist is more than just helping youth find a job or an education. It also includes addressing their emotional needs by normalizing failure and congratulating them throughout the job process, encouraging them to choose their own direction, and supporting them with instrumental activities of daily living to enable employment, such as taking the bus and learning time management–ultimately teaching skills for them to succeed not just in work, but in life. Several participants described that sometimes they are one of the few trusted adults in a youth’s life:

“I’ll laugh sometimes and say is it okay if I put my mom hat on… if you show respect and warmth, very often, you'll get it returned. And they know that I care. And it could be that, or may not be the first person that has shown that they cared in a very long time.” –P8 [SIC]

Making connections with youth, colleagues, and employers was a common theme throughout the data. Participants highlighted the importance of building relationships with youth over time so they can develop trust in employment specialists. Participants also described the need to make connections and build trust with potential employers, as “who you know” heavily impacts employment opportunities for their clients. Participants highlighted the need for more time in their roles to build relationships with employers and learn what resources are available in the community:

“It comes with having the time to be able to do that too. Quite often my day is incredibly like… right, as soon as I get here, I'm slammed and to be able to go out and network… It's a gift, and it's not often built into the work that we do […] So for us, it's a challenge to be able to and a luxury really to be able to leave and go out to community connections if we do not already have this in place.” –P13

## Discussion

Our study aimed to understand the unique strengths and significant barriers of providing employment services in rural northern communities. Employment specialists working in Northern BC communities operate at the intersection of macro-, meso-, and micro-level systems to support youth navigating employment and educational challenges, often alongside mental health and/or substance use concerns.

Our results align with literature that explores IPS-based services for youth aged 16–30 experiencing mental health challenges [19, 20]. Ellison [19] noted that there are complexities in providing employment and education services to this age demographic, including youth-centered care in navigating the transition to adulthood, supporting youth through the development of mental health and life skills, and engaging with employers–these concerns are also captured within our themes. Furthermore, our data highlight critical IPS components, including worker preferences, integrated services, and systematic job development, which require focused implementation efforts for this specific population [7].

Notably, our results indicate that youth in rural northern communities rarely seek employment services with “employment” as their sole presenting concern. Rather, employment services are most effective when integrated with broader health and social supports that address the complex and intersecting needs of young people. Employment specialists in our study emphasized needing dedicated time and resources to cultivate meaningful community and employer relationships. While these elements are fundamental to the IPS model, our findings suggest that their full realization remains challenging in resource-constrained and geographically dispersed settings such as Northern BC. Importantly, this also raises a broader question regarding the contextual fit of IPS: although IPS is an evidence-based model with strong empirical support, its assumptions regarding service intensity, integration, and rapid job development may not be equally feasible across all service environments, particularly those characterized by small, heterogeneous populations, limited workforce capacity, and competing service demands. This highlights the need to consider not only how IPS is implemented, but also where and under what conditions it may require substantial adaptation or complementary approaches.

This finding has global relevance, as youth in rural and underserved regions worldwide often face intersecting challenges related to employment, mental health, and social support. Implementing integrated, relationship-based employment services in low-resource settings is a common struggle internationally, highlighting the need for adaptable, context-driven models across diverse geographies [21, 22].

As IPS expands into varied service settings and populations, it is crucial for communities to carefully assess how adaptations impact fidelity and employment outcomes. We observed variation in the reported use of formal frameworks, which may reflect meaningful differences in how employment supports are structured and operationalized across rural service contexts. While a small number of participants explicitly identified the IPS model as a guiding framework, others described drawing on broader principles such as strengths-based practice and a focus on competitive employment, and several reported not working within any formalized model. This variation may reflect both the absence of standardized implementation structures in rural settings and the need for locally responsive practice adaptations.

Previous research confirms the efficacy of IPS in rural areas, but implementation fidelity is often compromised in under-resourced environments [8, 23]. To address this, systematic documentation, evaluation, and assessment of adaptations are essential [24]. Importantly, many IPS models and related supported employment frameworks were not originally designed for Northern, rural, or Indigenous community contexts. As such, effective implementation requires intentional engagement with local communities, including youth, families, Indigenous leaders, and service providers, to ensure that program delivery reflects local priorities, cultural values, and available resources. Our study, alongside existing literature, therefore, highlights a clear need for a rural, Northern-specific strategy to implement sustained SE programming that considers community context, youth needs, workforce capacity, and system capacity. Furthermore, implementation science frameworks should be used to research the impact, such as the Consolidated Framework for Implementation Research (CFIR) or Reach, Effectiveness, Adoption, Implementation and Maintenance (RE-AIM) [25, 26].

### Implications

This research highlights actions that employment specialists, employers, families, and policymakers can take to support sustainable SE programs in rural northern communities. At the macro level, understanding the evolving values and employment goals of youth within local labor markets is essential. At the meso level, strengthening relationships with employers can reduce stigma, support accommodations, and align youth and employer needs, with wage subsidies helping to reduce hiring barriers. At the micro level, recognizing that mental health needs may take priority and integrating mental health support within SE services are critical. Resourcing evidence-based models, such as the IPS and integrated youth services, may address the key barriers identified by participants, including the need for client-centered care, integrated service support, and time to build employer partnerships, ultimately improving support for youth.

### Limitations

This study has several limitations. Although the sample size of 10 employment specialists was appropriate for an in-depth qualitative study, it limits the breadth of perspectives and the generalizability of findings. Given the diversity of rural and remote communities across Canada and the limited understanding of the employment specialist workforce in these settings [27], the findings should be interpreted as illustrative rather than representative. While we achieved sufficient thematic saturation for this exploratory study, broader sampling across regions, service models, and workforce compositions may reveal additional insights.

The majority of participants identified as white, despite approximately 20% of the northern BC population identifying as Indigenous [14]. Future research should prioritize meaningful partnerships with Indigenous organizations and Elders from the outset to ensure culturally safe, trauma-informed approaches to supported employment. Further research should also explore the perspectives of youth and employers to better understand the barriers to and facilitators of implementing supported employment in rural and remote communities.

### Conclusion

Our findings highlight the need for context-sensitive implementation of supported employment in rural and northern communities, particularly for youth with mental health challenges. While these communities offer strengths such as strong relationships, resilient youth, and career opportunities, implementation must address barriers including limited access to mental healthcare, education, and transportation, stigma, inflexible employers, and fragmented services. Future implementation efforts should be co-designed with communities and build on their existing local strengths to ensure that programs are culturally responsive and sustainable.
