# Supplementary material for: “Work does change lives”: a qualitative study exploring employment specialists’ perspectives on assisting youth through supported employment in rural Canada
Source: Int J Public Health. 2026 Aug 27;71:1609182. doi: 10.3389/ijph.2026.1609182 (PMC13558120; doi:10.3389/ijph.2026.1609182)
Supplement: Supplementary file 1 [file Supplementaryfile1.docx]

**Supplemental File 1. Interview Questions**

**Employment specialists semi-structured interview questions**

1. Describe your role and experience in youth supported employment / education.

- In what ways have you interacted with supported employment programs?
- As a service provider, what is your role?
- How would you describe your role in supporting youth?
- What frameworks do you use within your role?

2. How would you describe the values of the region(s) you provide services in?

3. How are the values embodied in the services you provide to youth 12-24?

- If not, could you expand on why?

4. Recognizing there are challenges in transportation, intergenerational trauma, stigma, and structural challenges, what are the challenges youth face who are not engaged in employment or education?

- What are their barriers?

5. How do you support the youth facing those challenges?

6. What are the barriers to you supporting the youth?

- What supports do you need to better support the youth?

7. What adaptations would you like to see to remove those barriers?

8. How would you describe your relationship with the businesses in your area?

- How about education communities in your area?
- How does that relationship impact supporting youth find work / education?
- How do you foster that relationship?

9. What do you wish businesses and education centres knew or valued that would help you support youth?

- Or help youth in finding meaningful employment / study?

10. How would you describe your relationship with youth who are interested in work or education supports?

- What are the challenges?

11. Do you feel that your work is siloed or interconnected with other health services (i.e. counseling, social work, nursing)?

- How so?
- How can this be better?

12. Is there something we have not discussed which you want to bring up?

13. How would you like to see this data given back to you and your community?
